# Animal models on HTLV-1 and related viruses: what did we learn?

**DOI:** 10.3389/fmicb.2012.00333

**Published:** 2012-09-21

**Authors:** Hiba El Hajj, Rihab Nasr, Youmna Kfoury, Zeina Dassouki, Roudaina Nasser, Ghada Kchour, Olivier Hermine, Hugues de Thé, Ali Bazarbachi

**Affiliations:** ^1^Department of Internal Medicine, Faculty of Medicine, American University of BeirutBeirut, Lebanon; ^2^Service d’hématologie, Equipe labellisée, Ligue Nationale Contre le Cancer, CNRS/Université Paris Descartes Unité Mixte de Recherche 8147, Hôpital Necker–Enfants MaladesParis, France; ^3^Service de Biochimie, Equipe labellisée, Ligue Nationale Contre le Cancer, CNRS/INSERM/Université Paris Diderot Unité Mixte de Recherche 7212, Unité 944, Hôpital Saint LouisParis, France

**Keywords:** HTLV-1, BLV, STLV-1, animal models, ATL

## Abstract

Retroviruses are associated with a wide variety of diseases, including immunological, neurological disorders, and different forms of cancer. Among retroviruses, Oncovirinae regroup according to their genetic structure and sequence, several related viruses such as human T-cell lymphotropic viruses types 1 and 2 (HTLV-1 and HTLV-2), simian T cell lymphotropic viruses types 1 and 2 (STLV-1 and STLV-2), and bovine leukemia virus (BLV). As in many diseases, animal models provide a useful tool for the studies of pathogenesis, treatment, and prevention. In the current review, an overview on different animal models used in the study of these viruses will be provided. A specific attention will be given to the HTLV-1 virus which is the causative agent of adult T-cell leukemia/lymphoma (ATL) but also of a number of inflammatory diseases regrouping the HTLV-associated myelopathy/tropical spastic paraparesis (HAM/TSP), infective dermatitis and some lung inflammatory diseases. Among these models, rabbits, monkeys but also rats provide an excellent *in vivo* tool for early HTLV-1 viral infection and transmission as well as the induced host immune response against the virus. But ideally, mice remain the most efficient method of studying human afflictions. Genetically altered mice including both transgenic and knockout mice, offer important models to test the role of specific viral and host genes in the development of HTLV-1-associated leukemia. The development of different strains of immunodeficient mice strains (SCID, NOD, and NOG SCID mice) provide a useful and rapid tool of humanized and xenografted mice models, to test new drugs and targeted therapy against HTLV-1-associated leukemia, to identify leukemia stem cells candidates but also to study the innate immunity mediated by the virus. All together, these animal models have revolutionized the biology of retroviruses, their manipulation of host genes and more importantly the potential ways to either prevent their infection or to treat their associated diseases.

## INTRODUCTION

The family Retroviridae is composed of numerous non-icosahedral, enveloped viruses that possess two copies of a single-stranded RNA genome. The Retroviridae have two defining hallmarks of replication: the reverse transcription of the genomic RNA into a linear double-stranded DNA copy and the subsequent covalent integration of this DNA into the host genome. Among retroviruses, the Oncovirus family regroups many viruses having a clinical, economical, and veterinary significance. Human T-cell lymphotropic virus (HTLV)-1 belongs to the Delta-type retroviruses, which also include HTLV-2, -3, and -4, simian T-cell leukemia viruses STLV-1, -2, -3, -4, and -5, and bovine leukemia virus (BLV; **Table 1**). Animal models provide an excellent tool to understand the biology of oncoviruses related diseases, and to develop vaccines or targeted therapies. These animal models vary from naturally infected hosts to established or engineered animal models that mimic the related disease in patients (**Table 1**).

**Table 1:** BLV and PTLV: related hosts, diseases, and animal models.

Virus	Host	Disease	Animal models
Bovine leukemia virus (BLV)	Cattle	Leukosis	- Sheep - Rats - Rabbits
Human T-cell leukemia virus (HTLV-1)	Humans	- Adult T cell leukemia/lymphoma (ATL) - HTLV-1-associated myelopathy/tropical spastic paraparesis (HAM/TSP) - Infective dermatitis - Ocular lesions - Inflammatory arthropathy and polymyositis	- Primates - Rabbits - Rats - Mice models (xenografts, humanized, transgenics).
Simian T-cell leukemia virus (STLV)	Non-human primates	Unknown	Non-human primates

## NATURALLY INFECTED HOSTS

### BOVINE LEUKEMIA VIRUS

The symptoms of BLV were first discovered in 1871 when Leisering reported the occurrence in cattle of a disease called “leukosis” leading to splenomegaly associated with yellowish nodules in spleens of infected cows (Leisering, 1871). This spleen disruption is consecutive to tumor formation and is the most spectacular clinical manifestation in BLV infected cattle. Tumors result from accumulation of transformed B cells in the spleen as well as diverse organ infiltration of the liver, heart, eye, skin, lung, and lymph nodes (reviewed in Burny et al., 1987; Olson and Miller, 1987; Willems et al., 1999; Meas et al., 2002). This fatal lymphoma or lymphosarcoma occurs in <5–10% of infected animals, predominantly adult cattle older than 4–5 years (Ferrer, 1980; Burny et al., 1985, 1988) whereas the great majority of infected animals (around 70%) remain asymptomatic carriers of the virus. These animals can only be identified by the presence of anti-BLV antibodies and/or of proviral DNA (Kettmann et al., 1976; Burny et al., 1988; Kettmann and Burny, 1994). In these settings, <1 % of peripheral blood cells in animals are found to be infected by the virus (reviewed in Gillet et al., 2007). BLV can be transmitted though the milk horizontally (Ferrer and Piper, 1978). Nowadays, BLV causes major economical losses in cattle production and export (Trono et al., 2001; Motton and Buehring, 2003; Ott et al., 2003; Rhodes et al., 2003).

### PRIMATE T-CELL LYMPHOTROPIC VIRUSES: HUMAN T-CELL LEUKEMIA VIRUS AND SIMIAN T-CELL LEUKEMIA VIRUS

The primate T-cell lymphotropic viruses (PTLV) regroup the HTLVs (HTLV-1, -2, -3, and -4) as well as their related simian counterparts STLV-1, -2, and -3 (Lairmore and Franchini, 2007). Two additional STLV (-4 and -5) belong also to PTLVs but have no human counterparts discovered to date. While PTLV-1 and PTLV-2 strains have been extensively studied since the 1980s, studies on PTLV-3 are more recent and have increased in number since the discovery of HTLV-3 in 2005 (Calattini et al., 2005; Wolfe et al., 2005). HTLV-4, the fourth human HTLV retrovirus, was also discovered in 2005, but a simian counterpart of this virus has not been identified to date (Wolfe et al., 2005; Sintasath et al., 2009).

#### Simian T-cell leukemia viruses

The high percentage of homologies between HTLV and STLV strains, led to the demonstration that most HTLV subtypes arose from interspecies transmission between monkeys and humans. STLVs have been documented in more than 30 non-human primate (NHP) species from sub-Saharan Africa and Asia (Locatelli and Peeters, 2012). STLV-1 has been documented in captive but wild-caught chimpanzees and gorillas from west Central Africa (Gessain and Mahieux, 2000; Nerrienet et al., 2004). STLV-2 has only been documented in bonobos, an ape species endemic to Democratic Republic of Congo (Van Brussel et al., 1998). The first strain of STLV-3 was isolated in 1994, after the long-term co-culture of human cord blood lymphocytes with the peripheral blood mononuclear cells (PBMCs), obtained from an Eritrean sacred baboon that had been kept in captivity in a research laboratory in Leuven, Belgium (Goubau et al., 1994). Sequence comparisons of STLV-3 full-length proviruses pointed out that these strains are highly divergent from HTLV-1, HTLV-2, or STLV-2 prototype sequences (around 40% nucleotide divergence; Meertens et al., 2002, 2003; Meertens and Gessain, 2003).

#### Human T-cell leukemia viruses

HTLV-1 is the first human retrovirus discovered, and is the etiological agent of two distinct diseases: adult T-cell leukemia/lymphoma (ATL; Poiesz et al., 1980; Hinuma et al., 1981, 1982; Yoshida et al., 1982) and tropical spastic paraparesis/HTLV-1-associated myelopathy (TSP/HAM). ATL is an aggressive malignancy of mature activated CD4^+^ T cells, characterized by frequent visceral involvement, malignant hypercalcemia and opportunistic infections secondary to T cell immunosuppression. TSP/HAM is a slowly progressive neurodegenerative disorder in which lesions in the central nervous system (CNS) cause progressive weakness, stiffness, and a lower limb spastic paraparesis leading to the paralysis of the legs (Gessain et al., 1985; Rodgers-Johnson et al., 1985; Osame, 1986).

HTLV-1 infects approximately 20 million individuals worldwide (Matsuoka, 2003). Endemic areas include Japan, the Caribbean, inter-tropical Africa, Brazil, Eastern Europe, and the Middle East (Kaplan and Khabbaz, 1993; Abbaszadegan et al., 2003; Nagai and Osame, 2003). ATL develops in a small percentage (4%) of HTLV-1-infected individuals after a long period of clinical latency (20–40 years following viral infection; Hermine et al., 1998; Bazarbachi and Hermine, 2001; Bazarbachi et al., 2004). Yet, ATL is characterized by the monoclonal integration of HTLV-1 provirus in the tumor cells (Yoshida et al., 1984). Typical ATL cells are characterized by unusual morphology with lobulated nucleus, known as “flower cells” (Shimoyama et al., 1983). These malignant lymphocytes are activated CD4^+^ T cells with increased expression of the alpha chain of the interleukin (IL)-2 receptor (Waldmann et al., 1985; Okayama et al., 1997).

**FIGURE 1:**
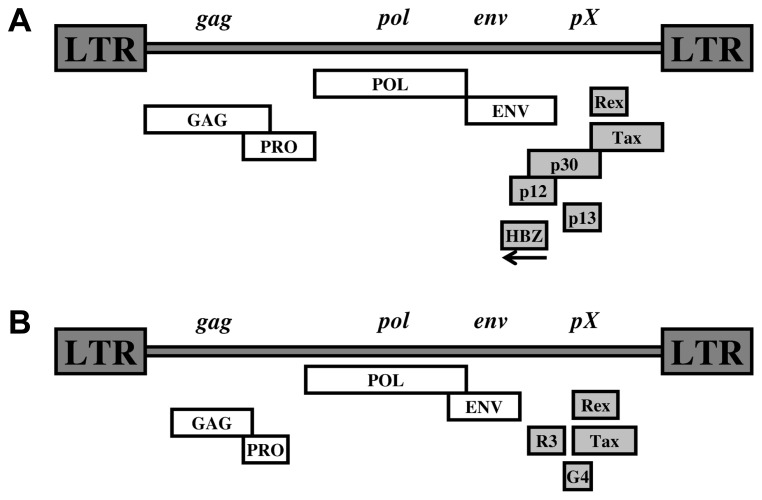
**(A)** HTLV-1 proviral genone. **(B)** BLV-1 proviral genome.

In addition to the classical structural genes required for retroviral replication, the HTLV-1 genome encodes a series of accessory and regulatory proteins (**Figure 1A**) such as the viral transcriptional activator Tax (Slamon et al., 1984) and the HTLV-1 bZIP factor gene (HBZ), a recently discovered unique viral protein encoded from the 3′ LTR in the complementary strand of the proviral genome (Gaudray et al., 2002). Both Tax and HBZ were shown to be linked to HTLV-1 pathogenesis (Boxus and Willems, 2009; Kannian and Green, 2010).

The diversity in prognosis and clinical features of patients with ATL led to the Shimoyama classification of this disease into four clinical subtypes: acute, lymphoma, chronic, and smoldering forms (Shimoyama, 1991). This classification is largely based on the extent of systemic leukemia, hypercalcemia, and organ involvement. The chronic and smoldering subtypes are considered indolent, but eventually have poor long-term survival. The acute and lymphoma forms generally have a worse prognosis mainly due to their resistance to the conventional chemotherapies, a large tumor burden, hypercalcemia, and/or frequent infectious complications as a result of a profound T-cell immunodeficiency (Shimoyama, 1991; Hermine et al., 1998; Bazarbachi and Hermine, 2001; Bazarbachi et al., 2004).

HTLV-1 is transmitted by three routes: (1) vertical (mother-to-child through breast-feeding), (2) horizontal (sexual), and (3) parenteral (blood transfusion or intravenous drug abuse) routes.* Via* any of the routes, infected cells are essential for transmission of HTLV-1, which has been demonstrated by the absence of seroconverters among recipients of fresh frozen plasma transfusions (Okochi et al., 1984).

A genetically related virus, HTLV-2, has been identified and isolated (Kalyanaraman et al., 1982). However, there has been no demonstration of a definitive etiological role for HTLV-2 in a human disease to date. HTLV-2 was originally identified from a patient with a variant form of hairy T-cell leukemia (Kalyanaraman et al., 1982) and is so far loosely correlated with TSP/HAM (Murphy et al., 1997a) or other opportunistic infections attributable to immunocompromised patients (Murphy et al., 1997b).

In 2005, the discovery of HTLV-3, a third HTLV type, was reported in two Cameroonese asymptomatic individuals living in the rainforest area of the southern part of the country (Calattini et al., 2005; Wolfe et al., 2005; Mahieux and Gessain, 2011). The fourth HTLV type (HTLV-4) was also reported in 2005 and consists only, so far, of a unique human strain, whose provirus was also found in the blood of a hunter living in Cameroon (Calattini et al., 2005). However, association of HTLV-3 and -4 with any human disease remains unconfirmed due to the limited number of cases in which these viruses have been identified (Thomas et al., 2010; Zheng, 2010; Mahieux and Gessain, 2011; Welsh, 2011).

## EXPERIMENTAL ANIMAL MODELS

### ANIMAL MODELS FOR BLV

#### Sheep are excellent models to study and follow BLV infection

Large animals often provide a more relevant model of human cancer as compared to mice, since disease chronology and relevant physiology are more accurately replicated. BLV transmission has been reported in rabbits (Wyatt et al., 1989; Onuma et al., 1990), rats (Altanerova et al., 1989; Boris-Lawrie et al., 1997), chicken (Altanerova et al., 1990), pigs (Mammerickx et al., 1981), and goats (Olson et al., 1981). However, the most consistent model to study BLV infection is the sheep (Djilali et al., 1987; Djilali and Parodi, 1989; Zhao et al., 2005).

Although BLV-associated ovine leukemia is a B-cell malignancy, it shares many similarities with ATL and has been extensively studied as a model for unraveling leukemogenic mechanisms (Willems et al., 2000; Gillet et al., 2007; Merimi et al., 2009). Since the complete onset of the disease occurs in a relatively short period of time (18 months average), this model was used for studying anti-leukemic immune responses prior to tumor onset. In addition, this model has been extensively used to develop potential treatment or vaccine against BLV infection in cattle as well as to correlate these approaches with HTLV-1-associated ATL.

As for the other complex retroviruses, in addition to the Gag, Pol, and Env structural and enzymatic proteins, the genome of BLV encodes essential regulatory and accessory proteins such as Tax and Rex (**Figure 1B**).

Malignant progression following BLV infection in sheep is dependent on the viral Tax oncoprotein (Yoshida et al., 1982; Burny et al., 1987; Willems et al., 1990; Schwartz and Levy, 1994; Marriott et al., 2002; Szynal et al., 2003; Jeang et al., 2004; Yoshida, 2005; Klener et al., 2006; Matsuoka and Jeang, 2007). Van den Broeke et al. (2010) developed a retrovirus transduction system to generate autologous B-cell lines expressing Tax. This group demonstrated that the induction of a Tax-specific cytotoxic response by DNA immunization or viral infection of naïve animals was not predictive of disease outcome and did not prevent tumor development. On the other hand, Florins et al. (2009) demonstrated that the integrity of the spleen is required to control pathogenesis because asplenia decreased the efficiency of the immune response and induced an imbalance in cell dynamics resulting in accelerated onset of leukemia.

Bovine leukemia virus-infected sheep were also used to provide insights on the molecular genetic and epigenetic modulation of viral expression. On the genetic level, infectious proviruses were cloned and injected into sheep or calves to study the viral genetic determinants required for infection and pathogenesis (Rovnak et al., 1993; Adam et al., 1994; Bartoe et al., 2000; Tana et al., 2001). One proviral clone (clone 344) leads to tumor or leukemia after a mean latency period of 33 months (Lefèbvre et al., 2002). This clone has been used to construct a series of derivative proviruses harboring mutations or deletions in different parts of the genome including *gag*, *pol*, or *env *genes whose deletions destroy infectivity *in vivo *(Onuma et al., 1987; Adam et al., 1994). The deletion of the region which expands from the end of the *env *gene to the splice acceptor site of the *tax/rex *mRNA does not impair infectivity (Adam et al., 1994). Since these sequences correspond respectively to the third and second exons of the R3 and G4 mRNAs, it appears that these genes are not essential for infectivity *in vivo*. When p12I and p13II/p30II orthologs of R3 and G4 were deleted, similar results were obtained (Collins et al., 1998; Van den Broeke et al., 2001; Silverman et al., 2004). Importantly, the R3/G4 deletion greatly interferes with the efficiency of BLV propagation and restricts pathogenesis (Powers et al., 1991; Lefèbvre et al., 2002). However, one out of 20 sheep infected with a R3/G4 mutant developed a lymphoma after 7.5 years of latency, demonstrating that the deleted sequences are not strictly required for pathogenesis (Florins et al., 2007). Among other isolates, clone 395 is deficient for infectivity *in vivo*, due to the presence of a mutation at codon 303 of the Tax protein (Adam et al., 1994; Tajima et al., 1998; Twizere et al., 2003). This result illustrates that Tax transactivation activity is required for viral infectivity *in vivo*. In contrast, a provirus (Tax106+293) harboring mutated phosphorylation sites remains infectious and propagates at wild-type levels in sheep. In addition, the Tax106+293 mutant is pathogenic despite a loss in its ability to transform primary cells *in vitro* (Szynal et al., 2003). The BLV transcriptional promoter located in the 5′ LTR contains suboptimal binding sequences for the CREB transcription factor. Remarkably, the cyclic-AMP responsive site (CRE) consensus “TGACGTCA” is never strictly conserved. When a perfect CRE sequence is restored, the promoter’s activity increases. However, the proviral loads are drastically reduced in sheep infected with a virus harboring this type of change (Calomme et al., 2004).

On the epigenetics level, a subtle equilibrium between the virus, which attempts to replicate, and the immune response, which seeks to exert tight control of the pathogen appeared to be tightly regulated by histone acetylation and DNA hypermethylation. In BLV infected cells, the virus is stably integrated apparently in a transcriptionally silent state (Kettmann et al., 1980; Gupta and Ferrer, 1982; Kashmiri et al., 1985; Van den Broeke et al., 1988; Lagarias and Radke, 1989; Kerkhofs et al., 1996; Merimi et al., 2007a,b). Two epigenetic mechanisms, histone acetylation and DNA hypermethylation, correlate with BLV transcriptional repression (Merezak et al., 2002; Tajima et al., 2003; Calomme et al., 2004; Nguy^e^n et al., 2004; Achachi et al., 2005; Pierard et al., 2010). A key observation in the BLV sheep model was a paradoxical decrease in proviral loads when increasing the BLV promoter efficiency (Merezak et al., 2001). This process was highly modulated by epigenetic modifications on the promoter sequence. In this context, a therapeutic approach based on the modulation of host epigenetic mechanisms was proposed to treat BLV infection and disease (Grange et al., 2000; Novakovic et al., 2004; Achachi et al., 2005). Different histone deacetylase (HDAC) inhibitors including valproate (VPA), trichostatin A (TSA), and trapoxin (TPX) efficiently enhanced viral transcription directed by the BLV promoter *in vitro* (Merezak et al., 2002; Achachi et al., 2005). HDAC inhibitors also increased viral expression during *ex vivo *short-term culture of PBMCs from BLV-infected sheep and cattle (Merezak et al., 2002; Achachi et al., 2005). VPA-induced hyperacetylation of histone H3 (Bouzar et al., 2009) and in the absence of any other cytotoxic drug, VPA-induced tumor regression in BLV-infected sheep. However, this therapy was inefficient for preventing primary infection or reducing proviral load in asymptomatic sheep (Achachi et al., 2005).

The BLV-infected sheep model was also used to unravel the relative importance of cell proliferation versus apoptosis during the process of leukemogenesis associated with infection by complex oncoviruses. Debacq et al. (2002) measured the rates of cell proliferation and death in the BLV-ovine system, by using the i.v. injection of 5-bromodeoxyuridine into BLV-infected sheep. Their results showed that the increase in the number of B cells during BLV-induced lymphocytosis results from an increased cell proliferation rather than a reduced cell death.

#### Rats and rabbits provide a tool for BLV vaccination

Ideally, the optimal vaccine shall contain a large number of viral factors permanently stimulating the immune response. Attenuated derivatives of BLV proviruses meet these requirements (Willems et al., 1993, 1997, 2000; Boris-Lawrie et al., 1997; Kucerova et al., 1999; Kerkhofs et al., 2000; Altanerova et al., 2004; Debacq et al., 2004; Florins et al., 2007). Replication-competent BLV proviruses lacking accessory genes and *cis*-acting LTR sequences were designed and evaluated in rats and rabbits. A first generation of these genetically simpler viruses was constructed by co-injection of independent vectors encoding *gag*-*pol *and *env *genes. These constructs were devoid of *tax*, *rex*, *R3*, and *G4 *and contained promoter *cis*-acting regulatory sequences of spleen necrosis virus (SNV). These BLV simpler hybrid derivatives were infectious and induced specific antibodies in a rat model (Boris-Lawrie et al., 1997). A second type of virus contained *gag*, *pol*, and *env *genes in a single genome under the control of SNV regulatory sequences. This viral vector was competent for replication and induced antibody responses against *gag *and *env *structural proteins in rats and rabbits (Boris-Lawrie et al., 1997; Kucerova et al., 1999; Altanerova et al., 2004). This viral vector induced protection against viral challenge in a rabbit model and decreased the proviral load (Altanerova et al., 2004).

### ANIMAL MODELS FOR PTLV

#### Non-human primates are both natural hosts and experimental models for STLV infections

Various species of NHP serve as the natural hosts for at least six exogenous retroviruses, including gibbon ape leukemia virus (GaLV), simian sarcoma virus, simian immunodeficiency virus (SIV), STLV, simian type D retrovirus (SRV), and simian foamy virus (SFV; Lowenstine and Lerche, 1988). Asian monkeys of the genus *Macaca*, are natural hosts for three of these viruses (SRV, SFV, STLV; Lowenstine et al., 1986; Daniel et al., 1988). These Macaques were widely used in diverse studies including vaccination and toxicology against retroviruses.

Using the primate animal model, Dezzutti et al. (1987) challenged vaccinated pig-tailed macaques with the HTLV-1 subunit vaccine by an STLV-1-infected cell line. An antibody response developed to HTLV-1 and STLV-1 viral proteins recognizing both gag and env proteins. Importantly, mononuclear cells from immunized monkeys produced a greater cytotoxic activity demonstrating that the HTLV-1 subunit vaccine was successful in protecting the pig-tailed macaques from the STLV-1 infection.

The primate animal model was also used in order to investigate the mode of transmission of HTLV-1 by studying the transmission of its related STLV-1. This study consisted of breeding seronegative macaques females with seropositive males and showed that sexual contact is important in the transmission of STLV-1, but it may not be an efficient mode of viral infection (Lazo et al., 1994).

On the molecular level, since the expression of the HTLV-1 provirus is epigenetically regulated, and since the low level of viral expression is associated with proviral chromatin deacetylation and condensation (Ego et al., 2002; Lu et al., 2004), an STLV-1 model was of great importance to study these mechanisms *in vivo*. Indeed, approximately 3% of all HTLV-1 infected persons will develop TSP/HAM against which there is currently no efficient treatment. Differences between the immune systems of rodents and humans cannot be ignored, particularly in models of TSP/HAM, because immune-mediated mechanisms appear to contribute to its development (Moore et al., 1989; Levin et al., 1997). As a correlation exists between the proviral loads (PVL) and the clinical status of the carrier, it is thought that diminishing the PVL could prevent later occurrence of the disease. In order to decrease the PVL, the STLV-1 model consisted of baboons (*Papio* *papio*) that are naturally infected with this virus. Baboons constitute an interesting, but little-used, model of asymptomatic HTLV-1 infection (Wolfe et al., 2005). Indeed, their immune system is very similar to the humans and the animals are naturally infected with STLV-1, and some develop STLV-1-associated diseases, such as ATL (Allan et al., 2001). Afonso et al. (2010) conducted a study combining VPA and zidovudine (AZT) in a series of baboons. They showed that the VPA/AZT combination induced a strong decrease in the PVL, which correlated with an increase in the STLV-1-specific cytotoxic T-cell population.

Non-human primates were also used to test the hypothesis that coinfection with human immunodeficiency virus (HIV) and HTLV-1 or -2 accelerates progression to AIDS. Fultz et al. (1999) inoculated pig-tailed macaques with the simian counterparts, SIV and STLV. During 2 years of follow-up of singly and dually infected macaques, no differences in SIV burdens, onset of disease, or survival were detected. However, in the first coinfected macaque that died of AIDS (1 year after infection), >50% of CD4^+^ and CD8^+^ lymphocytes expressed CD25. On the basis of the low incidence of HTLV-1- and STLV-1-associated disease during natural infections, this early evidence of neoplastic disease was unexpected. In the same direction, Gordon et al. (2010) generated a coinfection animal model to investigate the effect of HTLV-2 on T-cell response and its impact on SIV. They found that inoculation of irradiated HTLV-2 cells in macaques elicited humoral and T-cell responses to HTLV-2 at both systemic and mucosal sites. Their data provided insights on the potential development of an attenuated HTLV-2-based vector vaccine for HIV-1.

#### Rabbits are excellent models to study the immunological response against HTLV-1

Haynes et al. (2010a) established a rabbit HTLV-1 infection model to study early spatial and temporal events of the viral infection. Twelve-week-old rabbits were injected intravenously with cell-associated HTLV-1. Blood and tissues were collected at defined intervals throughout the study to test the early spread of the infection. Antibody and hematologic responses were monitored throughout the infection. This group showed that intravenous infection with cell-associated HTLV-1 targets lymphocytes located in both primary lymphoid and gut-associated lymphoid compartments. A transient lymphocytosis that correlated with peak virus detection parameters was observed by 1 week postinfection, before returning to baseline levels, suggesting that HTLV-1 promotes lymphocyte proliferation preceding early viral spread in lymphoid compartments to establish and maintain persistent infection (Haynes et al., 2010a). Moreover, Haines et al. developed an oral model of HTLV-1 transmission in rabbits to allow testing of the mucosal microenvironment during the early stages of orally acquired HTLV-1 (Martin et al., 2011). Valeri et al. (2010) suggested that infection of dendritic cells might be required for the establishment and maintenance of HTLV-1 infection in primate species. This conclusion was reached after their interesting finding established after the ablation of p12, p30, and HBZ proteins. In fact, none of these proteins, when ablated, could affect viral infectivity in rabbits. Interestingly, in rabbits, only the absence of HBZ is associated with a consistent reduction in virus levels. In contrast, in macaques, the absence of HBZ or p30 was associated with reversion of the mutant virus to the wild-type genotype. The macaques exposed to the p12 knockout remained seronegative. Interestingly, p12 and p30 mutants were severely impaired in their ability to replicate in human dendritic cells (Valeri et al., 2010). Furthermore, since HTLV-1-infected patients treated with immunosuppressive drugs, typically for organ or bone marrow transplantation procedures, often exhibit an accelerated or altered course for the development of HTLV-1-associated diseases (Gout et al., 1990; Brezin et al., 1995; Tsukasaki et al., 1999; Tajima and Aida, 2000), the rabbit model was used to evaluate the effects of immune suppression on the early spread of HTLV-1 infection upon treatment with cyclosporine A. Haynes et al. (2010b) concluded that immunologic control during early virus exposure determines subsequent HTLV-1 spread and has important implications for therapeutic intervention strategies and the development of HTLV-1-associated diseases.

#### Rats provided new insights on the relationship of regulatory T cells and ATL

The relationship of ATL cells with regulatory T cells (Treg) was intensively studied in order to explain the reasons behind the immunodeficiency in ATL patients. Some ATL cells and HTLV-1-infected human cells express Foxp3 and related molecules, such as CTLA-4 and GITR (Karube et al., 2004; Kohno et al., 2005; Matsubara et al., 2005; Roncador et al., 2005). To analyze the contribution of Foxp3 and Treg associated molecules to the development of ATL in more detail, various rat models for HTLV-1 infection including inbred and immunocompromised rats were generated (Ohashi et al., 1999; Nomura et al., 2004). Shinagawa et al. constructed a transgenic rat expressing human CRM1 (hCRM1), a cellular cofactor of Rex, and demonstrated that T cells derived from transgenic rats allowed production of HTLV-1 as efficiently as human T cells (Hakata et al., 2001; Takayanagi et al., 2007; Martin et al., 2011). Their results suggest the presence of inhibitor(s) during the entry process in rat dendritic cells (Martin et al., 2011).

#### Mouse models: a breakthrough in HTLV studies

Small animal models are the most efficient method of studying human afflictions. This is particularly evident in the study of the human retroviruses, especially HTLV-1. Indeed, although simian models were very useful to elucidate the mechanisms of early infection and cell-to-cell transmission and to study antiviral immunological responses and potential vaccine development, they remain expensive and difficult to maintain. Mice provide a cost-effective and highly reproducible model to study factors related to ATL development and the preclinical efficacy of potential therapies. Transgenic mice have provided important insight into viral genes responsible for lymphocyte transformation. Expansion of various strains of immunodeficient mice has accelerated the testing of drugs and targeted therapy against ATL.

## XENOGRAFT MICE MODELS

### DEVELOPMENT OF IMMUNOCOMPROMIZED MICE STRAINS

Over the past two decades, the construction of humanized animal models through the transplantation and engraftment of human tissues or progenitor cells into immunocompromised mouse strains has allowed the development of a reconstituted human tissue scaffold in a small animal system. The first humanized mouse model was developed in 1983 through the discovery of the *scid* mutation in CB-17 *scid/scid *(SCID) mice (Bosma et al., 1983). This mouse contains a spontaneous non-sense mutation in the gene for the protein kinase DNA activated catalytic polypeptide (Pkrdc). The Pkrdc enzyme is necessary for the efficient recombination of the B- and T-cell receptors. Without this enzyme, mature B and T cells do not develop. The SCID mouse retains normal macrophage, antigen-presenting cell, and natural killer (NK) cell functions (Bosma et al., 1983). SCID mice are used extensively in human stem cell and tumor cell engraftment studies. This mouse model resulted in animals demonstrating improved engraftment efficiency and infectivity. The SCID/beige mouse (CB17.B6-Prkdcscid/Lystbg) is a double mutant mouse in which the SCID mutation is retained, but these mice have an additional beige mutation in the Lyst gene that results in altered lysosomal trafficking. These mice have defective B- and T-cell function, NK cell activity, and granulocyte properties.

Engraftment efficiency was further improved through the integration of the non-obese diabetic (NOD) mutation leading to the creation of NOD/SCID, a good model used to study the development of autoimmune-mediated insulin-dependent diabetes mellitus. The resultant NOD/SCID mice lack functional B and T cells, have low NK cell activity, lack complement activity, and have impaired macrophage and antigen-presenting cell function. Other immunodeficient models were also created including NOD/SCID β2-microglobulin^null^ animals. These later were produced with development of the NOD/SCID mouse containing a targeted mutation in the β-2 microglobulin gene, encoding a protein necessary for the presentation of antigens *via* major histocompatibility class I. These mice lack all the immune functions that their less immunodeficient NOD/SCID predecessors also lack but have more complete elimination of NK-cell function.

Further efforts at minimizing the immune response resulted in the generation of NOG (NOD/Shi-*scid IL*2*r*γ^– / –^) mice. These mice are homozygous for the SCID mutation and a targeted disruption of the IL-2Rγ gene mutation. The γ chain is common to the receptors for IL-2, IL-4, IL-7, IL-9, IL-15, and IL-21. NOG mice are easily transplanted with human cells that would not normally transplant with the same efficiency in the more immunocompetent mouse models. NOG mice lack B- and T-cell development as well as NK-cell function and have a severe reduction in interferon (IFN)-γ production from dendritic cells. In order to further reduce the innate murine immune system, the Rag2^– / –^γc^– / –^ model was generated and constituted an important advancement for the engraftment of human CD34^+^ hematopoietic stem cells. These mutant mice were created by crossing homozygous recombinase activating gene 2 (Rag2) knockout mice with homozygous common cytokine receptor γ chain (γc) knockouts. The Rag2 mutation results in the lack of maturation of thymus derived T cells and peripheral B cells whereas the γc mutation results in the lack of the functional subunit of the IL-2, IL-4, IL-7, IL-9, and IL-15 receptors, preventing the development of lymphocytes and NK cells. The Rag2 knockout is not a leaky mutation: it does not result in spontaneously forming tumors, and does not confer radiation-sensitivity to the mice as the SCID mutation does. Therefore, the Rag2^– / –^γc^– / –^ mouse may be an ideal scaffold for repopulation of the animal with human hematopoietic cells (Greiner et al., 1995; Hesselton et al., 1995; Christianson et al., 1997; Ito et al., 2002; Ishikawa et al., 2005; Shultz et al., 2005; Ito et al., 2008; Pearson et al., 2008). Together, these animal models have revolutionized the investigation of retroviral infections *in vivo*.

### XENOGRAFTS OF HTLV-1 TRANSFORMED OR ATL CELLS IN IMMUNE-COMPROMIZED MICE

Xenografts of ATL cells or cell lines into immunodeficient mice replicate features of ATL and provide systems to test therapies (Zimmerman et al., 2010). Early attempts to establish an HTLV-1 infection *in vivo* involved inoculation of the CB17-*scid *mice with peripheral blood lymphocytes or PBMCs from HTLV-1 healthy carriers. These experiments were promising although limited in success due to engraftment inefficiencies and poor detection of viral integration (Feuer et al., 1993; Kondo et al., 1993). Feuer et al. (1996) used the SCID mouse model to compare the engraftment achieved with either HTLV-1-infected human hematopoietic progenitor CD34^+^ cells or *in vitro* HTLV-1**transformed cell lines SLB-1 and MT-2. This group showed that not only human hematopoietic progenitor cells could be infected *via* co-culture with cell lines transformed with HTLV-1 and HTLV-2, but that upon inoculation into immunocompromised mice, infection could be detected in biopsies from the thymus or the liver. When the same model was challenged using only the transformed cell lines SLB-1 and MT-2, infection could be also be detected in biopsies from the same organs, although levels were not as impressive as those achieved with the hematopoietic progenitor cells. These results pointed to a role for hematopoietic cells in HTLV-1 infection (Feuer et al., 1996).

To improve the efficacy of engraftment, Liu et al. (2002) used different HTLV-1 infected cell lines (RV-ATL cells) derived from a patient sample. This group noted that a higher level of engraftment could be achieved through the use of an HTLV-1 transformed cell line as opposed to cell lines that were immortalized through transfection, which did not produce lymphomas in NOD/SCID animals. These cells were reported to establish tumors readily, but must be propagated through mice because they did not remain viable in cell culture (Liu et al., 2002). This RV-ATL cell line was reported to engraft in approximately 75% of the SCID/beige mice, whereas transformed cells (HT-1-RV, SLB-1, MT-2, ACH, and ACH.p12) were unable to establish engraftment (Liu et al., 2002). These results illustrate the significant difference between ATL cell lines derived from patients versus those transformed *ex vivo* by HTLV-1. Furthermore, using C3H/HEJ model inoculated with MT-2 cells, Tanaka et al. (2001) demonstrated integration of the virus and concentration of infected cells in lymphoid tissue.

Kawano et al. (2005) developed a novel xenogeneic engraftment model in which primary ATL cells are transplanted intravenously into neonatal NOG SCID mice. Engrafted ATL cells were dually positive for human CD4 and CD25, and displayed patterns of HTLV-1 integration identical to those of donors. Engrafted mice showed monoclonal or polyclonal proliferation of ATL cells in blood and lymph nodes, evidenced by clinical features specific to each subtype of transplanted ATL.

### TARGETING THE NF-κB PATHWAY IN XENOGRAFT MODELS

Nuclear factor-κB (NF-κB) is a transcription factor constitutively activated in HTLV-1 infected and ATL cells (reviewed in Kfoury et al., 2005). NF-κB regulates the expression of a wide variety of genes implicated in proliferation, angiogenesis, invasion, and metastasis. Importantly, HTLV-1-induced transformation is dependent on the NF-κB activation, which makes this pathway an ideal target for therapeutic attack.

In tissue culture and mouse models, non-specific inhibitors of the NF-κB pathway such as sodium salicylate or cyclopentenone prostaglandins can increase the sensitivity of Tax-tumor cells to apoptosis and repress NF-κB-inducible cytokines IL-6, IL-10, IL-15, and IFN-γ (Portis et al., 2001). The proteasome inhibitor bortezomib is another non-specific inhibitor of the NF-κB pathway that is capable of inhibiting proliferation of ATL cells *ex vivo *and sensitizing them to apoptosis (Nasr et al., 2004; Mitra-Kaushik et al., 2004). Bortezomib inhibits the degradation of the NF-κB inhibitor IκBα, resulting in reversal of NF-κB activation. Hence, bortezomib treatment slowed tumor growth in an allograft model of ATL by increasing apoptosis, but toxicity constraints limited its efficacy (Mitra-Kaushik et al., 2004). Similarly, when bortezomib was administered into SCID mice bearing tumors, it suppressed tumor growth *in vivo*; confirming that bortezomib was effective against ATL cells *in vivo* (Satou et al., 2004).

Another inhibitor of NF-κB DNA binding activity, BAY 11-7082, was also shown to induce tumor regression in ATL transplanted NOG mice (Dewan et al., 2003). Similarly, Ohsugi et al. (2006), 2007 explored the use of the NF-κB inhibitor dehydroxymethylepoxyquinomicin (DHMEQ) as a therapeutic agent. They established a model for infection in the NOD/SCID β2-microglobulin null mice by sublethally irradiating 7–10-week-old animals and injecting them with transformed HTLV-1 cell lines the following day. Treatment with DHMEQ showed increased survival and growth inhibition of ATL cells in animals that had been infected through inoculation with HTLV-1 producing cell lines.

Finally, under a similar aim in targeting and understanding the NF-κB involvement *in vivo*, Nitta et al. (2008) utilized a mouse model with a defect in NF-κB inducing kinase (NIK) gene resulting in a phenotype of alymphoplasia (*aly*/*aly*). These investigators used this model to evaluate the importance of NIK for the establishment of HTLV-1 infection and associated pathology. *Aly*/*aly *mice were compared with C57BL/6J and BALB/c mice. All animals were inoculated intra-peritoneally with MT-2 cells, and PCR was used to evaluate PVL. *Aly*/*aly *animals demonstrated dramatically lower PVLs, suggesting that NIK plays an essential role in HTLV-1 infection and could serve as a potential target for therapeutic intervention (Nitta et al., 2008). Altogether, these results confirm the importance of NF-κB activation in ATL development and demonstrate that NF-κB inhibition can slow ATL growth, but is not sufficient for ATL eradication.

### TESTING MONOCLONAL ANTIBODIES IN XENOGRAFT MICE MODELS

The expression of markers on the cell surface of ATL cells implanted in mice has made them an excellent target for preclinical trials with monoclonal antibodies. Monoclonal antibodies directed against IL-2Rα (Phillips et al., 2000), CD25, CD52, and CD2 (Zhang et al., 2003a,b, 2005) were tested. Phillips et al. (2000) established a NOD/SCID animal model by introducing cells from an ATL patient (MET-1), which are activated T cells that express CD2, CD3, CD4, CD25, CD122, and CD52, into the mice. The disease progressed to death in this animal model after approximately 4–6 weeks. When they treated the animals with humanized anti-Tac (HAT), murine anti-Tac (MAT), and 7G7/B6, all of which are directed to CD25 (IL-2Rα), they noticed that all of the treatments significantly delayed the progression of the leukemia and prolonged the survival of the tumor-bearing mice. Moreover, and using the same animal model, Zhang et al. (2005) investigated the therapeutic efficacy of flavopiridol, an inhibitor of cyclin-dependent kinases, alone and in combination with HAT. They obtained a prolonged survival and a dramatically enhanced antitumor effect, with the combination therapy. Zhang et al. (2005) evaluated the efficacy of Campath-1H (alemtuzumab; a humanized monoclonal antibody directed to CD52), alone and in combination with HAT or with MEDI-507 directed to CD2. They noticed that the survival of the group receiving the Campath-1H was significantly longer than that of the group receiving the HAT. Furthermore, the main tumor killing mechanism with Campath-1H *in vivo* involves FcRgamma-containing receptors (e.g., FcRgammaIII) on polymorphonuclear leukocytes and macrophages that mediate antibody-dependent cellular cytotoxicity and/or trigger cross-linking induced apoptosis (Zhang et al., 2003a,b). The outcome of these mouse studies may be predictive of successful therapy for human patients, because a complete response has been reported in an ATL patient treated with alemtuzumab (anti-CD52; Mone et al., 2005). Maeda et al. (2010) investigated the effect of CD30-mediated therapy on ATL by using SGN-30, a chimeric anti-CD30 mAb, and SGN-35, a monomethyl auristatin E-conjugated anti-CD30 mAb, *in vitro* and *in vivo*. They used NOD/SCID mice subcutaneously engrafted with HTLV-1-infected cell lines. Both mAbs significantly inhibited the growth of HTLV-1-infected cell tumors in NOD/SCID xenograft models, suggesting that CD30-mediated therapy with SGN-30 or SGN-35 would be useful for patients with ATL.

## HUMANIZED MICE MODELS

Miyazato et al. (2006) took the next step in 2006 when they designed an experiment utilizing the NOG mouse model. Their investigation involved inoculation with human PBMCs in order to establish a humanized system, followed by inoculation with the MT-2 cell line to allow for the required cell-to-cell transmission essential for HTLV-1 infection. Important findings included the detection of an increased PVL in both CD4^+^ and CD8^+^ T cells. Additionally, they were able to demonstrate that prophylaxis with the reverse transcriptase inhibitors tenofovir and azidothymidine (AZT) was successful in preventing new HTLV-1 infection in these animals. Takajo et al. (2007) were able to achieve similar results in 2007 when they also established HTLV-1 infection in NOG mice through the inoculation of PBMCs from HTLV-1-infected individuals. Although the approach was different, they confirmed that these animals could harbor HTLV-1 infection and they demonstrated the presence of detectable viral integration.

Another attempt to explore treatment options included a novel approach to detect tumor growth. Shu et al. (2007) established a bioluminescent mouse model in the older CB17-*scid *model by infecting the animals with the ATL cell line, RV-ATL, and a lentivirus harboring the luciferase gene. These investigators were able to non-invasively measure the tumor growth and expansion that occurred in the recipient mice. Additionally, they tested a bisphosphonate, zoledronic acid, and the proteasome inhibitor bortezomib. Both compounds demonstrated some level of success in reducing the development of tumors, as well as levels of parathyroid hormone related protein (PTHrP) and macrophage inflammatory protein-1α (MIP-1α) which are both indicators of malignant hypercalcemia, a complication observed in 60% of acute ATL patients (Shu et al., 2007).

Chen et al. (2009) utilized the NOD/SCID mouse inoculated with an ATL cell line, MET-1, in their investigation of the use of a HDAC inhibitor, depsipeptide, along with daclizumab as a therapeutic option in the murine HTLV-1 infection model. They demonstrated that both depsipeptide and daclizumab alone and when used in combination were able to increase the survival of the animals.

### ROLE OF HEMATOPOIETIC STEM CELLS IN HTLV-1 HUMANIZED MICE MODELS

In order to identify the molecular and cellular events that control the initiation and progression of ATL and potential therapeutic targets to block tumor development, Banerjee et al. (2010) generated an HTLV-1-infected humanized (HU-NOD/SCID) mouse model. This model was obtained by inoculation of NOD/SCID mice with CD34^+^ hematopoietic progenitor and stem cells (CD34^+^ HP/HSCs) infected *ex vivo* with HTLV-1. HTLV-1-HU-NOD/SCID mice exclusively developed CD4^+^ T-cell lymphomas with characteristics similar to ATL. Importantly, an increased proliferation of infected human stem cells (CD34^+^CD38^-^) in the bone marrow was observed in mice developing malignancies. Furthermore, CD34^+^ HP/HSCs purified from the PBMCs of an HTLV-1-infected patient revealed proviral integrations suggesting viral infection of human bone marrow-derived stem cells. NOD/SCID mice reconstituted with CD34^+^ HP/HSCs transduced with a lentivirus vector expressing the HTLV-1 oncoprotein Tax also developed CD4^+^ lymphomas. The recapitulation of a CD4^+^ T-cell lymphoma in HU-NOD/SCID mice suggests that HSCs provide a viral reservoir *in vivo* and act as cellular targets for cell transformation in humans.

Tezuka et al. reported the development of ATL-like disease in humanized mice (huNOG) by the intra-bone marrow transplantation of NOG-SCID mouse with CD133^+^ hematopoietic stem cells purified from human cord blood infected with HTLV-1 (Martin et al., 2011). Inverse PCR analysis of provirus integration sites revealed oligoclonal expansion of infected T cells in CD4^+^/CD25^+^ T cells similar to HTLV-1-infected humans. Villaudy et al. (2011) reported that HTLV-1 induces alterations of the thymus of Rag2^-^/IL-2R γc^-^ mice leading to expanded populations of mature CD4^+^/CD25^+^ T cells and other pathological features such as splenomegaly and lymphomas as compared to mock-infected mice. This unique model system was then used to test anti-cancer drugs, further illustrating the usefulness of the model (Martin et al., 2011; Villaudy et al., 2011).

### ROLE OF THE IMMUNE SYSTEM IN HTLV-1 INFECTION AND ATL DEVELOPMENT

In order to understand the immune response against HTLV-1 in infected patients, xenografted mouse models were of great importance. Stewart et al. (1996) demonstrated that SCID mice NK cells mediated specific lysis of HTLV-1-expressing cell lines, suggesting that the absence of HTLV-1 expression in patient-derived ATL lines allows these cells to evade immune surveillance. Whole-body irradiation or administration of antibodies to abrogate NK-cell function proved necessary to establish engraftment of non-leukemic cell lines such as SLB-1 cells (Feuer et al., 1995; Uchiyama, 1996; Liu et al., 2002). MT-2 cells developed tumors at the site of injection in SCID mice treated with anti-asialo GM-1 antibody, which functionally inactivates NK cells (Ishihara et al., 1992). Indeed, invariant NK T cells (iNKT) are inversely correlated to PVLs in ATL patients (Azakami et al., 2009).

## TRANSGENIC MICE MODELS OF HTLV-1

Transgenic animal technology has been useful for the direct demonstration of the tumorigenic potential of oncogenes *in vivo*. Over the recent years, a wide variety of oncogenes and proto-oncogenes from viral and cellular sources have been inserted into the germline of mice with subsequent development of neoplasia. These models continue to provide new insights into the molecular mechanisms of HTLV-1-associated transformation. None of the transgenic mice models fully recapitulate HTLV-1-associated disease, but many have been useful to investigate Tax-mediated disruption of lymphocyte function or provide evidence that Tax is an oncoprotein.

### THE HTLV-1 ONCOPROTEIN Tax

In addition to its effects on the transactivation of the viral LTR, Tax has pleiotropic cellular functions (Franchini, 1995; Gatza et al., 2003; Matsuoka, 2003). It stimulates the transcription of several cellular genes through activation of critical transcription factors such as NF-κB (Sun et al., 1994; Good and Sun, 1996; Uhlik et al., 1998; Xiao et al., 2001); cyclic AMP response element-binding protein (CREB; Zhao and Giam, 1992; Suzuki et al., 1993), serum responsive factor (SRF; Fujii et al., 1992), and activated protein 1 (AP-1; Fujii et al., 2000). Tax also represses the expression of cellular genes such as DNA polymerase β (Jeang et al., 1990), cyclin A (Kibler and Jeang, 2001), and transforming growth factor β (Arnulf et al., 2002). Moreover, Tax is involved in the regulation of apoptosis through the activation of apoptosis-suppressing genes such as Bcl-XL (Nicot et al., 2000) and repression of apoptosis-inducing genes such as Bax (Brauweiler et al., 1997). Tax inhibits tumor suppressor proteins such as p53 and p16, interferes with cell cycle checkpoint control and enhances the accumulation of mutations in HTLV-1-infected cells through the repression of DNA repair (Suzuki and Yoshida, 1997; Pise-Masison et al., 1998). Tax also influences the microenvironment: it induces angiogenesis and gap junction mediated communication between infected cells and endothelial cells, hence contributing to the extravasation and invasiveness of ATL cells (El-Sabban et al., 2002; Bazarbachi et al., 2004). Recent observations suggested that Tax also modulates the micro-RNA environment thereby adding another level of complexity to its cellular functions (Jeang, 2010). Indeed, a recent study (Yamagishi et al., 2012) has shown that Mi-RNA31 negatively down-regulates the non-canonical NF-κB pathway by targeting NIK. Aberrant up-regulation of polycomb proteins contribute to miR-31 down-regulation epigenetically leading to activation of NF-κB and apoptosis resistance in ATL cells (Yamagishi et al., 2012). Furthermore, more than 20 cellular proteins have been reported to interact with Tax, including a number of cytoplasmic proteins, such as MEKK1, MAD1, CBP, RelA, and IκB kinase subunits, as well as other nuclear proteins that are not found in Tax Speckled Structures (TSS), including p16^INK4a^ and p15^INK4b^ (Jin et al., 1998; Yin et al., 1998; Xiao et al., 2000; Azran et al., 2005). Interactions of Tax with these proteins have profound effects on normal host cell processes and in many cases have been shown to be essential for or to enhance cellular transformation.

Among the properties of Tax, activation of the NF-κB pathway plays a crucial role in the proliferation and transformation of HTLV-1-infected T cells (Yamaoka et al., 1996; Robek and Ratner, 1999). In unstimulated cells, NF-κB is found in an inactive cytosolic complex, associated with IκB. Upon cell stimulation, the IκB proteins are phosphorylated by the IκB kinase (IKK) complex, then ubiquitylated and subsequently degraded by the proteasome. Consequently, RelA-containing NF-κB proteins translocate to the nucleus, bind specific promoters, and activate NF-κB-dependent gene transcription (Li and Gaynor, 2000). Tax acts at multiple levels to initiate and maintain a permanent NF-κB activation (reviewed in Geleziunas et al., 1998; Sun et al., 2000; Jeang et al., 2004). A critical step is the recruitment of Tax to the IKK-γ regulatory component of the IKK complex (Yamaoka et al., 1998). Tax/IKKγ association leads to activation of the IKKα and IKKβ kinases resulting in IκB phosphorylation, ubiquitylation, and proteasomal degradation. The precise subcellular localization where these events occur and their molecular requirements remain largely unknown. We and others recently demonstrated that NF-κB activation is dependent on Tax post-translational modifications, namely ubiquitylation and sumoylation (Lamsoul et al., 2005; Nasr et al., 2006; Kfoury et al., 2008, 2011, 2012), which result in the activation of the IKK complex, phosphorylation of the NF-κB inhibitor IκB, ultimately resulting in the nuclear translocation of the active NF-κB subunits and activation of NF-κB-dependent genes (reviewed in Kfoury et al., 2005, 2012). In addition, Peloponese et al. (2004) reported that ubiquitin addition modifies Tax in a proteasome-independent manner from an active to a less-active transcriptional form. Finally, Tax subcellular distribution and its interaction with cellular proteins respond dynamically to cellular stress (Gatza and Marriott, 2006).

Altogether, these multiple activities of Tax cooperate to promote infected T-cell proliferation, generate cellular defects and lead to subsequent transformation. This oncogenic potential of Tax was demonstrated through its ability to transform a rat fibroblast cell line (Matsumoto et al., 1997) and immortalize primary T cells *in vitro* (Grassmann et al., 1992).

### Tax TRANSGENIC MICE MODELS

Tax in transgenic mice models is sufficient to cause oncogenesis. However, leukemia and lymphoma are rare. The first transgenic mice expressing the HTLV-1 tax gene (originally called HTLV-1 tat when mice were produced) under the control of the LTR promoter [Tg (HIV-tat) 6-2Gja] developed multicentric mesenchymal tumors of the nose, ear, mouth, tail, and foot (Nerenberg et al., 1987). These tumors were characterized by a typical spindle cell component with infiltration of granulocytes. Although this transgenic system is not appropriate for the study of ATL, it proved that the tax gene encodes an oncoprotein. In addition to the mesenchymal tumors described above, these transgenic mice developed a disease characterized by degeneration of oxidative muscle fibers. As such, this transgenic mouse helped to understand some aspects of HTLV-1-associated myopathies (Nerenberg and Wiley, 1989). LTR-tax mice showed skeletal abnormalities. Bones were grossly thicker and more fragile, whereas histologically they exhibited high bone turnover characterized by increases in osteoclasts and osteoblasts (Ruddle et al., 1993). Combining the LTR-tax mice with LTR-βgal (β galactosidase) mice generated a bitransgenic mouse in which the transactivator protein acts on the LTR to increase expression of βgal. The enzyme was detected in bone, muscle, cartilage, exocrine glands, and mesenchymal tumors (Benvenisty et al., 1992). Recently, Swaims et al. (2010) focused on the role of HTLV-1 expression in chronically infected CD4^+^ T cells using LTR-Tax transgenic mice. In this system, immune activated Tax-expressing CD4^+^ T cells express characteristics of several different CD4^+^ T cell subtypes, suggesting that HTLV-1 Tax induces changes in the normal pattern of CD4^+^ subtype specification (Kress et al., 2011).

Several transgenic C57/CBA mouse strains were generated with tax gene under the regulatory control of CD-3 promoter enhancer sequence designed to target expression to leukocytes. These mice developed mesenchymal tumors at wound sites as well as mammary and salivary adenomas (Hall et al., 1998). Another model of Tax transgenics was developed in C57BL/6TgN mice (huGMZBTax) under the control of the granzyme B promoter. In this model, Tax expression was restricted to CD4^+^, CD8^+^, NK cells, and lymphokine-activated killer cells (Grossman et al., 1995). These mice exhibit a large granular lymphocytic (LGL) leukemia and neutrophilic dominated inflammation at sites of trauma admixed with LGL leukemic cells. These mice also developed splenomegaly, lymphadenopathy, and masses on the ears, legs, and tail (Grossman et al., 1995). As in ATL patients, these mice had malignant hypercalcemia and osteolytic bone lesions associated with metastasis (Gao et al., 2005). This model was also used to study the contribution of p53 inactivation to Tax-mediated tumorigenesis (Portis et al., 2001). Primary Tax-induced tumors and tumor-derived cell lines exhibited functional inactivation of the p53 apoptotic pathway and were resistant to an apoptosis-inducing stimulus. In contrast, *p53 *mutations in tumors were found to be associated with secondary organ infiltration. Furthermore, mating Tax transgenic mice with p53-deficient mice demonstrated minimal acceleration in initial tumor formation, but significantly accelerated disease progression and death in mice heterozygous for *p53* suggesting that functional inactivation of p53 by HTLV-1 Tax, is not critical for initial tumor formation, but contributes to late-stage tumor progression (Portis et al., 2001). Using the same transgenic model, Rauch et al. (2009) reported that Tax expression in IL-15 knockout mice led to the development of larger and more aggressive tumors, suggesting caution against IL-15 blockade as an ATL therapy (Martin et al., 2011).

An advantageous model to study the role of inflammation and its relationship with tumor development was obtained when the C57BL/6TgN (huGMZBTax) were crossed with an IFN-γ knockout strain. The obtained mouse model showed an enhanced rate of lesion development (Mitra-Kaushik et al., 2004). More exploitations were done on imaging tumor engraftment *in vivo*. Indeed, Rauch et al. (2009) evaluated Tax-mediated activation of luciferase in LTR-luciferase (LTR-LUC) mice [C57BL/6TgN(LtrLuc)] refining the C57BL/6TgN mouse model. They reported that microscopic intraepithelial lesions precede the onset of peripheral subcutaneous tumors and that Tax is sufficient for inducing tumors. These results suggest that the viral oncoprotein activates lymphocytes to cause NK/T-cell recruitment, activation, and subsequent transformation.

In order to more specifically target Tax expression to leukocytes, bitransgenic doxycycline inducible mice [Tg (EmuSR-tTa) 83Bop] were generated to specifically control expression of wild-type or selected tax mutants in the lymphocyte compartment. This model showed skin manifestations as in ATL patients with a fatal dermatologic disease characterized by infiltration of Tax-positive T cells into the dermis and epidermis. Addition of doxycycline (suppression of Tax expression) resulted in the resolution of lesions (Kwon et al., 2005).

Hasegawa et al. (2006) generated tax transgenic mice in which the transgene expression was restricted to the thymus by the Lck promoter [C57BL/6-Tg (Lck-HTLV-1 Tax)]. This mouse model was a further confirmation that Tax alone can induce leukemia and hence represents the powerful oncogene of HTLV-1 virus. Indeed, HTLV-1 Tax transgenic mice were generated using the *lck *proximal promoter to restrict transgene expression to developing thymocytes. Following prolonged latency periods (around 18 months), animals developed diffuse large cell lymphomas and leukemia with clinical, pathological and immunological features characteristic of acute ATL with characteristic flower cells, and extensive lymphomatous infiltration of the spleen, lymph nodes, bone marrow, liver, kidney, and lung by malignant T lymphocytes highly expressing CD25 (Hasegawa et al., 2006). As in ATL patients, mice showed marked leukocytosis, hypercalcemia, and high level of LDH and constitutive activation of the NF-κB pathway (Hasegawa et al., 2006; El Hajj et al., 2010).

### IDENTIFICATION OF ATL STEM CELLS IN MURINE ATL DERIVED FROM Tax TRANSGENICS

Transferring murine ATL splenic cells derived from Tax transgenics (Hasegawa et al., 2006) to NOD/SCID mice allowed the identification of the first candidate ATL stem cells in a side population (0.06%), which overlapped with a minor population of CD38^-^/CD71^-^/CD117^+^ cells (0.03%). In addition, lymphoma and ATL stem cells could also be demonstrated in the bone marrow and in both osteoblastic and vascular niches. In these ATL stem cells, *Tax*, *Notch1*, and *Bmi1 *expression was down-regulated, suggesting that they were derived from Pro-T cells or early hematopoietic progenitor cells (Yamazaki et al., 2009). Using the same Tax transgenic model, Kawaguchi et al. (2009) demonstrated that AMD3100, a CXCR4 antagonist, inhibited infiltration of lymphomatous cells into liver and lung tissues *in vivo*. Their results demonstrated the involvement of the stromal cell-derived factor-1α (SDF-1α) and its receptor CXCR4 interaction as one mechanism of leukemic cell migration and this may provide a novel target as part of combination therapy for ATL. The same tax transgenic mouse model generated by Hasegawa et al. (2006) was used in an interesting study using a bioinformatics approach where Suzuki et al. (1993) could identify in a comparative proteomic analysis, proteins differentially expressed in Tax induced lymphoma (Martin et al., 2011). Strikingly, among the more than 700 proteins detected, levels of 53 proteins were increased in stem cells, including one membrane protein, which might potentially serve as a new target of antibody based therapy (Martin et al., 2011).

### Tax TRANSGENICS AS A PLATEFORM TO TEST TARGETED THERAPY OF ATL

In addition to the xenograft humanized mouse models, transgenic mice provided insights to clinicians before the development of phase I clinical trials. They were used in many translational studies to examine the effect of different targeted therapies of ATL (summarized in Zimmerman et al., 2010).

Adult T-cell leukemia/lymphoma is resistant to chemotherapy and carries a very poor prognosis (reviewed in Shimoyama, 1991; Bazarbachi et al., 2004). Multiple small studies using AZT and IFN showed response in ATL patients. A worldwide meta-analysis recently showed that antiviral therapy significantly increased 5-year survival of ATL patients from 20 to 50% (Bazarbachi et al., 2010). Unfortunately, most patients eventually relapse, which underlines the need for new therapeutic approaches. Using an *in vitro* model of ATL derived cell lines and freshly isolated ATL leukemic cells, Bazarbachi et al. (1999) showed that arsenic trioxide synergizes with IFN to selectively induce G1 arrest and apoptosis in ATL cells. This combination yielded promising clinical results in relapsed/refractory ATL patients (Hermine et al., 2004). Critically, this drastic phenotype was associated to rapid proteasome-mediated Tax degradation upon exposure to the drug combination (El-Sabban et al., 2000; Nasr et al., 2003). Promising results were recently obtained in *de novo* ATL patients treated with arsenic trioxide, IFN, and AZT, with 100% response rate including 70% complete remission rate (Kchour et al., 2009).

El Hajj et al. (2010) recently reported that the combination of arsenic trioxide and IFN cures Tax-driven murine ATLs through selective targeting of leukemia initiating cell (LIC) activity. We used a transplantation model of murine ATL by transferring murine ATL splenic cells derived from Tax transgenics (Hasegawa et al., 2006) to NOD/SCID mice. These mice develop ATL-like disease manifested by diffuse large cell lymphomas and leukemia with clinical, pathological, and immunological features characteristic of acute ATL with typical flower cells, and extensive lymphomatous infiltration of the spleen, lymph nodes, bone marrow, liver, kidney, and lung by malignant T lymphocytes highly expressing CD25 (Hasegawa et al., 2006). As in ATL patients, mice showed marked leukocytosis, hypercalcemia, and high level of LDH and constitutive activation of the NF-κB pathway (Hasegawa et al., 2006; El Hajj et al., 2010). Unexpectedly, ATL cells did not respond to arsenic and IFN by apoptosis and/or cell cycle arrest *in vivo* and therefore this regimen does not induce rapid tumor regression or massive cell death in treated animals. Importantly, on the other hand, this combination therapy used in primary mice immediately reduces leukemia transplantation into untreated secondary recipients and totally abrogates leukemia transplantation into untreated tertiary recipients. In other words, the primary tumor continues to grow and only exhausts much later, due to the specific targeting of LIC activity. Adding the proteasome inhibitor bortezomib essentially blocks the degradation of Tax triggered by the arsenic/IFN combination, and eliminates the enhancement of survival in secondary and tertiary recipients. This reversal of ATL LIC eradication by proteasome inhibition is a significant indication that ATL cells are addicted to continuous Tax expression for their LIC activity (stemness) but not for their short-term tumor growth.

Since the action of the arsenic/IFN combination is very specific to both HTLV-1-infected cells and Tax-driven murine leukemia, it is most likely that therapy-induced loss of the driving oncogene underlies responsiveness to therapy.

### HBZ TRANSGENICS DEVELOP TUMORS BUT NOT ATL

Despite the fact that the expression of Tax is frequently disrupted in ATL (Matsuoka and Jeang, 2007), the HTLV-1 bZIP factor (HBZ) gene, which is encoded by the minus strand of the HTLV-1 genome (Larocca et al., 1989; Gaudray et al., 2002), is transcribed in all ATL cases (Satou et al., 2006). The HBZ gene product promotes the proliferation of ATL cells (Satou et al., 2006; Arnold et al., 2008). Furthermore, HBZ mRNA expression in HAM/TSP patients was well correlated with disease severity (Saito et al., 2009). Satou et al. (2011) generated transgenic mice containing the HBZ gene under control of a murine CD4-specific promoter/enhancer/silencer (HBZ-Tg mice). HBZ-Tg mice spontaneously developed systemic dermatitis, alveolitis, and lymphoma as they aged and expressed the HBZ gene in all murine CD4^+^ cells. This group demonstrated that transgenic expression of HBZ in CD4^+^ T cells induced T-cell lymphomas and systemic inflammation in mice. Importantly, whereas human ATL cells and murine ATL cells derived from tax transgenics displayed constitutive activation of the NF-κB pathway, NF-κB was not activated in T-cell lymphomas observed in HBZ transgenics, hence demonstrating that HBZ alone cannot maintain the ATL phenotype *in vivo*

Since the immune system plays a major role in HAM/TSP progression and since HBZ mRNA expression was correlated with disease severity (Saito et al., 2009), Satou et al. (2011) used the HBZ transgenic mouse model to study the CD4^+^Foxp3^+^ Treg population specifically that ATL cells were shown to functionally and phenotypically resemble Foxp3^+^CD25^+^CD4^+^ Treg cells, which control immune responses against self- and non-self-antigen (Sakaguchi et al., 2008). In HBZ-transgenic mice, CD4^+^Foxp3^+^ Treg cells and effector/memory CD4^+^ T cells increased *in vivo.* As a mechanism of increased Treg cells, HBZ expression directly induced Foxp3 gene transcription in T cells. However, the increased CD4^+^Foxp3^+^ Treg cells in HBZ transgenic mice were functionally impaired while their proliferation was enhanced (Satou et al., 2011). To further investigate the expression of Foxp3, Taguchi et al. used the HBZ transgenics to investigate the production of cytokines and they provided data to support the concept that altered Foxp3 expression in iTreg cells might result in systemic inflammation (Matsuoka and Green, 2009; Martin et al., 2011).

## CONCLUSION

Collectively, advances in the development of animal models have extended opportunities to better understand the biology of HTLV-1-related diseases. Rising from naturally infected to genetically engineered models, these animals provided new insights on the biology of HTLV-1 and related viruses. They allowed a better understanding of the multiple genetic, epigenetic, and cellular aberrations that occur during the progression of leukemia in humans, cattle, and monkeys. They highlighted the immune response against these viruses providing new insights on the potential development of vaccines. Humanized mice models using immunocompromised animals pointed to the importance of hematopoietic stem cells in HTLV-1 infection and ATL development. Genetically engineered mice demonstrated that Tax triggers ATL development, allowed the identification of potential ATL stem cells and the preclinical development of targeted therapies such as monoclonal antibodies or the combination of arsenic trioxide and IFN-α.

Using the translation of knowledge from the laboratory bench to appropriate animal models and subsequently to patients provides hope for the prevention, and/or development of targeted and efficacious treatments against a highly refractory neoplasm such as ATL. For the first time, a proposed drug regimen showed an efficient and specific targeting of the leukemia initiating cells and was translated into an excellent responsiveness of human patients yielding to improved survival and potential cure of ATL.

## Conflict of Interest Statement

The authors declare that the research was conducted in the absence of any commercial or financial relationships that could be construed as a potential conflict of interest.
